# Forcing neural progenitor cells to cycle is insufficient to alter cell-fate decision and timing of neuronal differentiation in the spinal cord

**DOI:** 10.1186/1749-8104-3-4

**Published:** 2008-02-13

**Authors:** Valérie Lobjois, Sophie Bel-Vialar, Françoise Trousse, Fabienne Pituello

**Affiliations:** 1 Centre de Biologie du Développement, UMR5547, Institut d'Exploration Fonctionnelle des Génomes IFR109, Université Toulouse III et Centre National de la Recherche Scientifique, 31062 Toulouse, France; 2 Laboratoire de Biologie Cellulaire et Moléculaire du Contrôle de la Prolifération, UMR5088, Institut d'Exploration Fonctionnelle des Génomes IFR109, Université Toulouse III et Centre National de la Recherche Scientifique, 31062 Toulouse, France; 3 Univ Montpellier 2, Montpellier, 34095 France; Inserm, U710, Montpellier, 34095 France; EPHE, Paris, 75007 France

## Abstract

**Background:**

During the development of the nervous system, neural progenitor cells can either stay in the pool of proliferating undifferentiated cells or exit the cell cycle and differentiate. Two main factors will determine the fate of a neural progenitor cell: its position within the neuroepithelium and the time at which the cell initiates differentiation. In this paper we investigated the importance of the timing of cell cycle exit on cell-fate decision by forcing neural progenitors to cycle and studying the consequences on specification and differentiation programs.

**Results:**

As a model, we chose the spinal progenitors of motor neurons (pMNs), which switch cell-fate from motor neurons to oligodendrocytes with time. To keep pMNs in the cell cycle, we forced the expression of G1-phase regulators, the D-type cyclins. We observed that keeping neural progenitor cells cycling is not sufficient to retain them in the progenitor domain (ventricular zone); transgenic cells instead migrate to the differentiating field (mantle zone) regardless of cell cycle exit. Cycling cells located in the mantle zone do not retain markers of neural progenitor cells such as Sox2 or Olig2 but upregulate transcription factors involved in motor neuron specification, including MNR2 and Islet1/2. These cycling cells also progress through neuronal differentiation to axonal extension. We also observed mitotic cells displaying all the features of differentiating motor neurons, including axonal projection via the ventral root. However, the rapid decrease observed in the proliferation rate of the transgenic motor neuron population suggests that they undergo only a limited number of divisions. Finally, quantification of the incidence of the phenotype in young and more mature neuroepithelium has allowed us to propose that once the transcriptional program assigning neural progenitor cells to a subtype of neurons is set up, transgenic cells progress in their program of differentiation regardless of cell cycle exit.

**Conclusion:**

Our findings indicate that maintaining neural progenitor cells in proliferation is insufficient to prevent differentiation or alter cell-fate choice. Furthermore, our results indicate that the programs of neuronal specification and differentiation are controlled independently of cell cycle exit.

## Background

Embryonic neural stem cells can either proliferate, thereby maintaining a pool of undifferentiated neural progenitor cells, or differentiate into neurons or macroglial cells. In the developing nervous system two principal factors determine the fate of the differentiating neurons or glia: the position of the neural progenitor cell within the neuroepithelium and the timing of initiation of its differentiation.

In the developing spinal cord, the ventricular zone contains neural progenitor cells that are subdivided into groups destined for distinct neuronal differentiation [1]. At early developmental stages, the ventral neural progenitor cells, termed progenitors of motor neurons (pMNs), can produce motor neurons, while at later stages they differentiate into oligodendrocytes. The pMNs express a unique combination of homeodomain transcription factors, leading to the upregulation of the basic helix-loop-helix (bHLH) transcription factor Olig2 [2-6]. Olig2 occupies a key nodal point in the pathway, contributing to the regulation of both homeodomain transcription factors, which determine motor neuron subtype specification, and bHLH factors, like the proneural factor neurogenin 2 (Ngn2), which drive neurogenesis. Oligodendrocyte production requires the ongoing activity of Olig2 and is preceded by downregulation of Ngn2, a determinant of the neuron-glial switch [7]. While oligodendrocytes retain the capacity to divide after leaving the neural progenitor domain, neuronal progenitor cells exit the cell cycle prior to initiating migration and differentiation in the mantle layer. Cell cycle exit represents part of the proneural activity of Ngn2 [3,4,8-10].

The impact of the timing of cell cycle exit on neural cell fate and the timing of neuronal differentiation remains unclear. Data indicate that cell cycle exit alone is insufficient to trigger neuronal differentiation [11,12]. Conversely, the onset of neuronal differentiation may be hindered by forcing neural progenitor cells to cycle [13]. D-type cyclins (CyclinDs) are known to govern progression in G1, and forced expression of CyclinDs at early stages of spinal cord development keeps neural progenitor cells proliferating, impeding neuronal differentiation [13]. In the hindbrain of *jumonji *(*jmj*) mutant mice, failure to turn off CyclinD1 alters the timing of neuronal differentiation. Although the cells migrate into the differentiating field, they retain neural progenitor traits, including the ability to divide. This phenotype is rescued by crossing the *jmj *mutant with CyclinD1 knockout mice [14]. Reports also exist in the literature of neuronal cells re-entering the cell cycle after migration and/or initiation of neuronal differentiation [10,15-17]. For example, in the developing spinal cord the cyclin-kinase inhibitor (CKI) p57kip2 is expressed transiently in the nuclei of nascent interneurons. In the absence of p57kip2, many interneurons re-enter the cell cycle inappropriately for at least one additional round of cell division but later exit to begin differentiation [10]. The authors proposed a requirement of the Cip/Kip subfamily of CKIs in timing neuronal cell cycle exit but not differentiation. To date, p27kip1 is the only CKI whose expression is described to be both initiated and maintained in the nuclei of nascent motor neurons; however, genetic disruption of p27kip1 causes no detectable neurogenic defect in the spinal cord [10]. Thus, the influence of the timing of cell cycle exit on motor neuron production remains difficult to determine.

To establish more clearly the importance of the temporal regulation of cell cycle exit on cell-fate choice, we wished to test the effect of forced cycling on the specification and timing of differentiation. To this end, we tested the fate of pMNs, which temporally result in two distinct cell types, motor neurons and oligodendrocytes, following the forced expression of CyclinD. We show that forcing pMNs to cycle does not alter the production of motor neurons but, rather, results in transgenic cells migrating to the differentiating field and differentiating whilst cycling. This leads to the generation of differentiated motor neurons that incorporate bromodeoxyuridine (BrdU) and express mitotic markers. Our findings demonstrate that retaining cells in the cell cycle is not sufficient to maintain a reservoir of undifferentiated neural progenitor cells, with the cycling cells instead proceeding with their programmed specification and differentiation, regardless of cell cycle exit.

## Results

### Long-term forced expression of CyclinD leads to the presence of proliferating cells in the differentiating field

We previously showed that forced expression of CyclinD1 or D2 for 24 hours in the neural tube favors the proliferation of transgenic neural progenitor cells at the expense of neuronal differentiation [13]. These results suggest that maintaining neural progenitor cells in proliferation is a means of keeping them undifferentiated in the ventricular zone.

**Figure 1:**
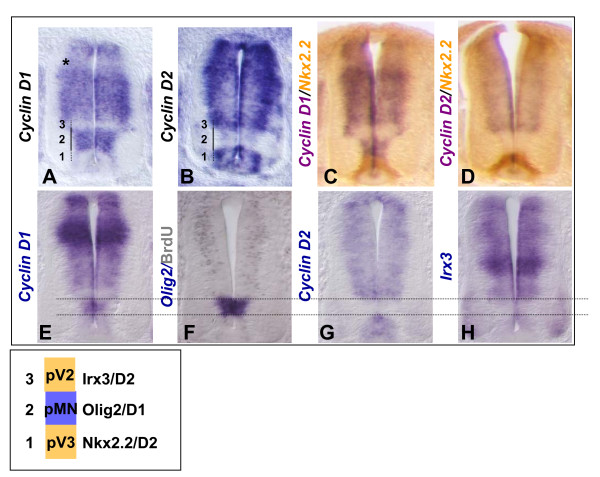
**D-type cyclins display discrete domains of expression in the developing spinal cord.(a,b) ***In situ *analysis of CyclinD1 (a) and D2 (b) expression on cross-sections of the neural tube of 3- to 3.5-day-old chicken neural tube. **(c,d) **Co-detection of the transcripts CyclinD1 or D2 (dark blue) with the protein Nkx2.2 (brown). **(e-h) ***In situ *hybridization performed on serial sections showing CyclinD1 (e), Olig2 in combination with anti-BrdU immunostaining (f), CyclinD2 (g), and Irx3 (h). Note that both CyclinD1 and D2 are present in the dorsal aspect of the ventricular zone (a,b). A small dorsal domain expresses CyclinD1 at a lower level (a, asterisk). In the ventral part of the spinal cord, distinct domains (1, 2, 3) of ventral progenitors express distinct CyclinDs, the pMN domain expressing mainly CyclinD1. Transcripts encoding CyclinD1 seem more homogenously distributed along the apico-basal axis than those encoding CyclinD2, which are reinforced on the basal side (b).

**Figure 2:**
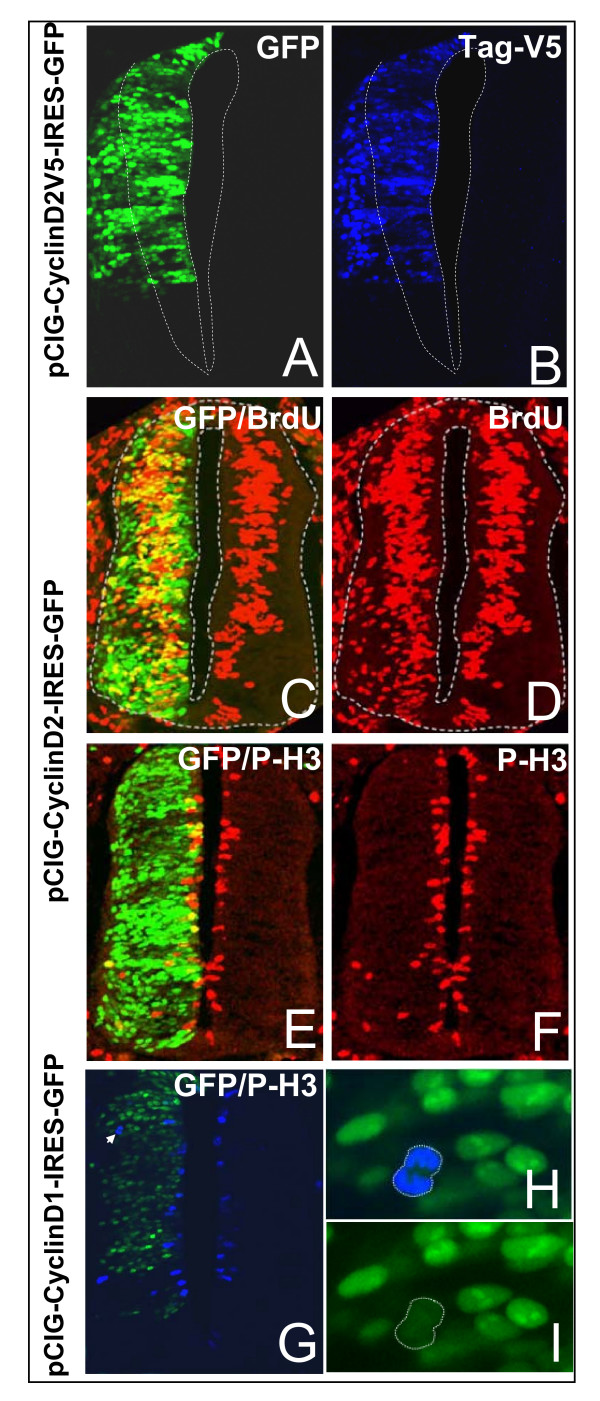
**Transgenic cells overexpressing CyclinD migrate into the differentiating field while remaining in the cell cycle.(a,b) **Forty-eight hours following forced expression of a tagged-CyclinD2 version, cells expressing GFP (green) and the transgenic protein detected with an anti-Tag-V5 antibody (blue) are observed in the ventricular zone and differentiating field. **(c,d) **Detection of the cells in S phase visualized following a 30 minute pulse of BrdU (red). **(e-i) **Detection of the phospho-histone H3 (P-H3) on a cross-section of the spinal cord 48 h after overexpression of CyclinD2 (e,f, red) or D1 (g-i, blue). (h,i) Magnifications of the cell in anaphase shown in (g) (arrowhead). (a-f) Maximum projections of eight optical sections acquired at 5 μm Z steps; (g-i) single optical sections.

To investigate this further, we analyzed the behavior of neural progenitor cells overexpressing CyclinD1 or CyclinD2 for longer periods of time to determine whether these cells remain as undifferentiated progenitors. CyclinD1-internal ribosomal entry site (IRES)-green fluorescent protein (GFP), CyclinD2-IRES-GFP or control IRES-GFP expression constructs were transfected by *in ovo *electroporation on the left side of the neural tube of 1.5-day-old chicken embryos [13]. The effects of CyclinD1 or D2 forced expression were analyzed at 48 hours after electroporation, that is, in 3- to 3.5-day-old spinal cord corresponding to a peak in motor neuron production. Cross-sections through the spinal cord of wild-type 3- to 3.5-day-old embryos showed that *CyclinD1 *and *D2 *transcripts were still expressed in the ventricular zone, the pMN domain specifically expressing CyclinD1 (Figure 1). Cross-sections of the CyclinD-electroporated spinal cord displayed GFP positive cells (GFP+) located in the mantle zone (Figure 2) that were still expressing CyclinD, as confirmed by means of a tagged version of CyclinD2 (pCIG-CyclinD2V5-IRES-GFP) (Figure 2a,b). This demonstrates that forced expression of CyclinD1 or D2 does not alter migration of the transgenic cells to the differentiating field.

To ascertain whether CyclinD transgenic cells located in the mantle layer were still cycling, we performed a 30 minute pulse of BrdU just before fixation of the embryos to detect cells in S phase, and immunostaining using phospho-histone 3 antibody (P-H3) to detect mitotic cells. In the wild-type spinal cord, or in embryos electroporated with a control vector (data not shown), cells in S phase were restricted to the ventricular zone and mitotic cells confined along the lumen of the neural tube (Figure 2c–g, right side). In the spinal cord of embryos electroporated with CyclinD1 (n = 5/5 embryos) or CyclinD2 (n = 1/1 embryo), we observed numerous GFP+ cells in S phase outside the ventricular zone (Figure 2d). This was not observed in embryos electroporated with a pCIG-IRES-GFP control vector (n = 0/5 embryos; data not shown). We also clearly detected ectopic P-H3 positive transgenic cells located in the differentiating field of embryos electroporated with CyclinD1 or D2 (n = 9/9 with CyclinD1 and 10/10 with CyclinD2; Figure 2e–i) but not in transgenic embryos electroporated with a pCIG control vector (n = 0/9 embryos; data not shown). We observed cells in anaphase/telophase among the ectopic P-H3 positive cells, suggesting a progression through metaphase (Figure 2h,i). These observations show that neural progenitor cells overexpressing CyclinD can migrate out of the ventricular zone despite being in the cell cycle. Hence, cell cycle exit is not a prerequisite for neural progenitor cell migration.

### Cycling neural progenitor cells in the mantle layer do not express markers of the ventricular zone

**Figure 3:**
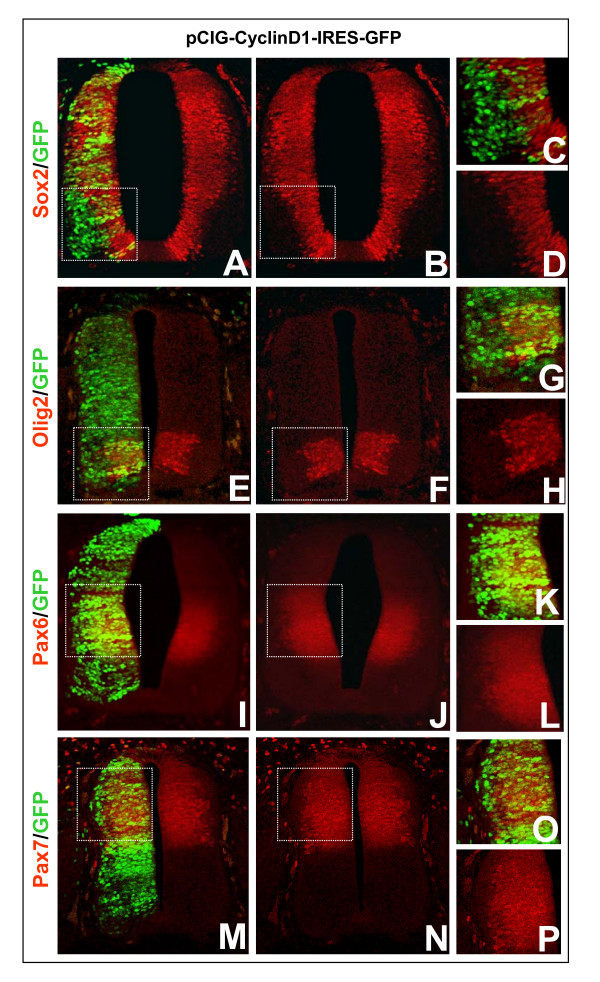
**Analysis of transcription factors restricted to neural progenitor cell domains in transgenic cells overexpressing CyclinD.** Immunodetection of **(a-d) **Sox2, **(e-h) **Olig2, **(i-l) **Pax6, and **(m-p) **Pax7, showing that transgenic cells (green) co-express markers of the ventricular zone (yellow cells) only when located within that zone. For each marker, a total of at least 400 transgenic cells were analyzed from 5 independent 40 μm sections from 4 transgenic embryos. Each image represents the maximum projections of 8 optical sections acquired at 5 μm Z steps. (c,d,g,h,k,l,o,p) High magnifications of the zones framed by dashed lines on the adjacent sections.

In the developing spinal cord, proliferating neural progenitor cells restricted to the ventricular zone express a specific panel of transcription factors, such as Sox2 throughout the entire zone and Olig2, Pax6 and Pax7 in discrete domains along the ventral to dorsal axis [1]. Expression of these proteins is switched off once cells migrate out of the ventricular zone and differentiate. Following our observations of transgenic cells overexpressing CyclinD1 or D2 proliferating in the mantle layer, we wanted to test whether these cells retain the expression of proteins normally restricted to the ventricular zone, namely Sox2, Olig2, Pax6 and Pax7. For each of these protein markers, we examined 400 transgenic cells located in the mantle layer. As shown in Figure 3, we detected no markers of the ventricular zone in cells overexpressing CyclinD1 in the mantle layer. These data show that transgenic cycling cells in the differentiating field do not retain the markers found in neural progenitor cells.

### Cycling cells progress normally in their program of specification

**Figure 4:**
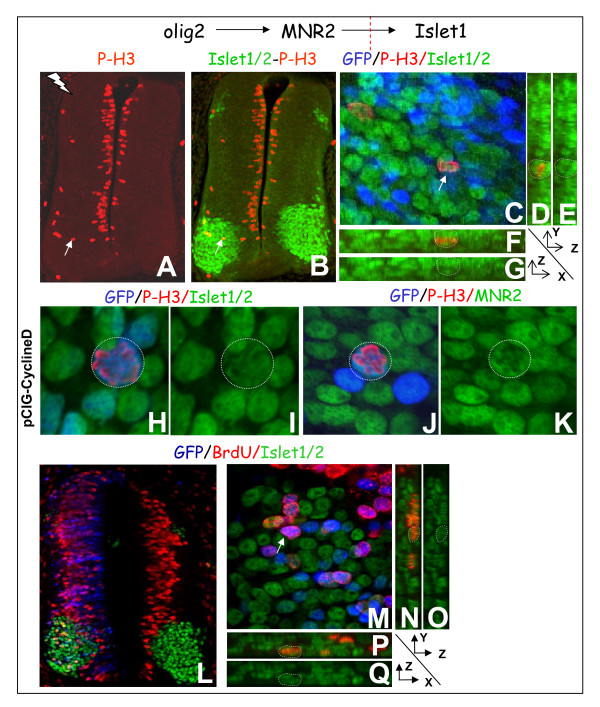
**Motor neurons progress in their program of specification while remaining in the cell cycle.(a-k) **Co-immunodetection of Islet1/2 (a-i, green) or MNR2 (j,k, green) with P-H3 (red). (a,b) Maximum projections; the electroporated side is on the left. (c) Single optical section showing a high magnification view at the level of the cell marked with an arrow in (a,b). (d-g) Orthogonal sections along the cell marked with an arrow in (c). (h-k) Single optical sections taken with a 63× objective. Dotted lines mark the limit of the mitotic transgenic cell. **(l-q) **Co-immunodetection of BrdU (red) and Islet1/2 (green). All the pictures are single optical sections. (n-q) Orthogonal projections along the cell marked with an arrow in (m).

Numerous changes occur at the gene expression level of neural progenitor cells committed to becoming post-mitotic neurons, including the onset of a program of specification. The well described sequence of activation of transcription factors involved in motor neuron specification includes an upregulation of MNR2 in committed motor neuron progenitors during their ultimate division and of Islet1 in post-mitotic differentiating motor neurons (Figure 4). We observed that all the transgenic cells located in the motor neuron columns and overexpressing CyclinD1 or D2 were Islet1/2+. In accordance with this, we identified the expression of MNR2 and Islet1/2 in ectopic P-H3-expressing cells in the mantle layer (Figure 4c–k). After a 30 minute pulse of BrdU, 32 ± 3.5% (standard error of the mean (SEM); n = 24 optical sections from 9 embryos electroporated with CyclinD1) of the transgenic Islet1/2+ cells were in S phase (Figure 4l–q). Such a proliferation rate is characteristic of a population proliferating rapidly and asynchronously. These data suggest that the sequence of activation of transcription factors involved in motor neuron specification is not altered in cycling pMNs.

### Cycling cells progress through differentiation to axonal extension

**Figure 5:**
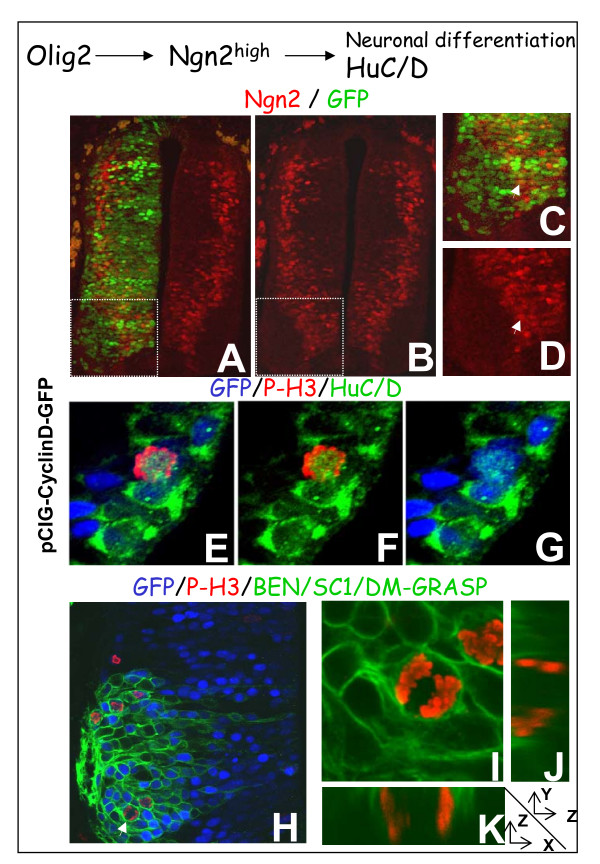
**Cell cycle exit is not required for initiation or progression of neuronal differentiation.(a-d) **Immunodetection of Ngn2 (red) after 48 h of CyclinD1 forced expression. The transgenic cells located in the motor neuron differentiating field do not retain Ngn2 expression. (a-d) maximum projections; (c,d) high magnifications of the zone in the dotted lines in (a) and (b), respectively. The arrow marks a transgenic cell expressing Ngn2. **(e-g) **Single optical sections showing the co-expression of HuC/D (green) and P-H3 (red) in the ventral horn of a spinal cord 48 h after electroporation with a CyclinD2 expression vector. **(h-k) **Co-immunodetection of BEN/SC1/DM-GRASP (green) and P-H3 (red) 48 h after electroporation with CyclinD1. (h-k) Single optical sections. (i-k) High magnifications of the cell marked with an arrow in (h); the GFP channel is off. (j,k) Orthogonal sections along the mitotic cell.

The progression of neurogenesis also depends on the ordered expression of transcription factors involved in generic neuronal differentiation, initiated by the selection of neural progenitor cells that upregulate the proneural Ngn2. Scattered upregulation of Ngn2 occurred in transgenic cells overexpressing CyclinD1 located in the lateral ventricular zone, similar to that seen on the control side (Figure 5a–d). Similarly, as with the other markers of the ventricular zone, Ngn2 was not detected in cells overexpressing CyclinD1 in the mantle layer. Instead, these cells expressed the pan-neuronal marker HuC/HuD, with transgenic cells expressing both P-H3 and HuC/HuD after electroporation of CyclinD1 (data not shown) or D2 (Figure 5e–g). As a complementary marker, we used the BEN/SC1/DM-GRASP antibody to recognize a membrane glycoprotein of the immunoglobulin superfamily on motor and sensory neurons as well as floor plate cells [18]. BEN/SC1/DM-GRASP is at first detected on the motor neurons at the level of the cell body just after withdrawal from the cell cycle and soon afterwards its location extends to the elongating axon. As illustrated in Figure 5h–k, after 48 h of CyclinD1-forced expression, we clearly observed transgenic mitotic cells expressing BEN/SC1/DM-GRASP at the level of the cell body. These observations further demonstrate the ability of transgenic cells to differentiate whilst remaining in the cell cycle.

**Figure 6:**
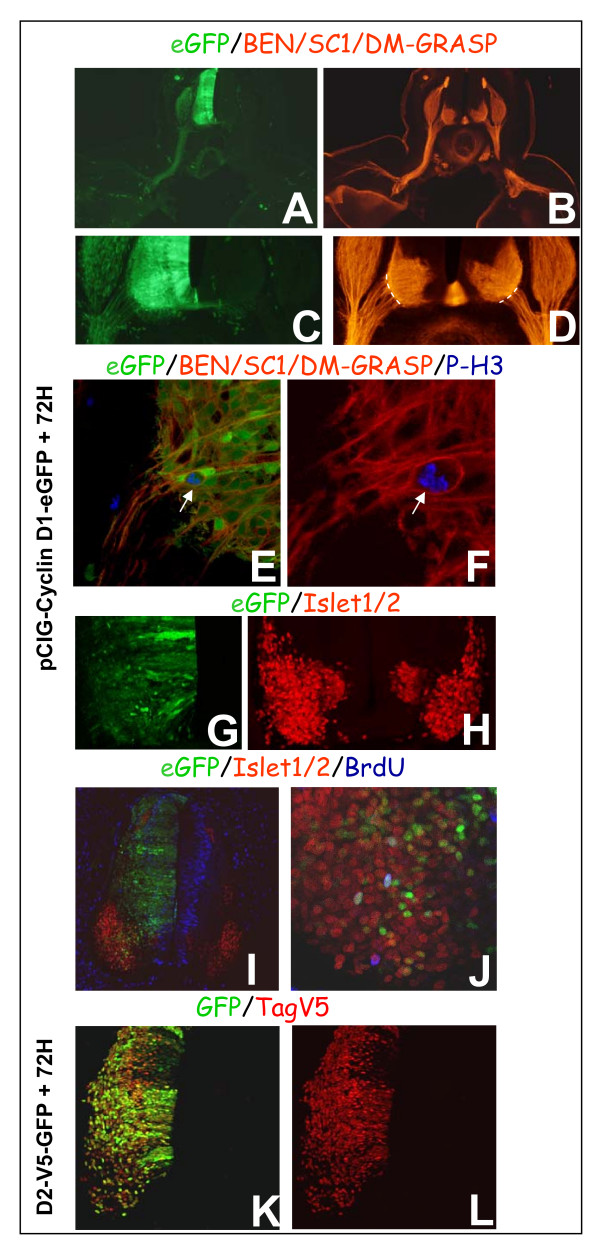
**Effects of 72 h of sustained expression of CyclinD on motor neuron differentiation.(a-d) **Transverse sections through the spinal cord at the brachial level showing the axonal tracts. eGFP+ axons are found in motor nerves, in the ventral commissure and in the sensory nerves, that is, corresponding to the tracts originating from the different locations where transgenic cells are detected. Immunodetection of BEN/SC1/DM-GRASP (red) reveals an enlarged ventral root on the electroporated side compared to the contralateral control side (underlined with dashed lines in (d)). (a,d) Images obtained with an epifluorescent microscope. **(e,f) **Co-immunodetection of BEN/SC1/DM-GRASP (red) and P-H3 (blue) 72 h after electroporation with CyclinD1 reveals the presence of mitotic cells displaying an axon. (e-f) Single optical sections; (f) high magnification of the cell marked with an arrow in (e) – the GFP channel is off. **(g,h) **Single optical sections showing eGFP and Islet1/2 (red) 72 h after CyclinD1 electroporation. The population of motor neurons is increased on the transgenic side. **(i,j) **Single optical sections showing eGFP, Islet1/2 (red) and BrdU (blue). (j) A high magnification of (i). **(k,l) **Seventy-two hours following forced expression of a tagged version of CyclinD2, cells expressing GFP (green) still express CyclinD2 visualized with an anti-Tag-V5 antibody (red). The TagV5 is clearly detected in the differentiating field. (k,l) Maximal projections.

To determine whether these cycling and differentiating motor neurons were able to extend axons, a CyclinD1-IRES-eGFP expression construct was created, enhanced GFP (eGFP) having previously been used successfully to follow axonal tracts [19]. This new construct resulted in a similar phenotype to that described with the CyclinD1-IRES-GFP construct (data not shown). We analyzed embryos 72 h after electroporation, a stage when axons are well developed. Figure 6a,c shows numerous eGFP+ axons exiting out of the spinal cord via the ventral root and projecting along nerve branches supplying the body wall and limb bud. Combining eGFP detection with BEN/SC1/DM-GRASP showed that these transgenic axons, emanating from the ventral root, originate from motor neurons (Figure 6a–d). Some of these transgenic cells extending BEN/SC1/DM-GRASP-positive axons also expressed the mitotic marker P-H3, confirming the axon-growing ability of a neuron even though in mitosis (Figure 6e,f). As electroporation allows only the transient expression of transgenes, we were unable to determine whether these motor neurons exhibit synaptic differentiation at more mature stages.

Together, these data show the ability of spinal progenitor cells to migrate, differentiate and extend axons independently of their cell cycle exit, arguing strongly against cell cycle exit representing a gate for neuronal differentiation.

### Sustained expression of CyclinD1 leads to an increase in the motor neuron population

Whilst we observed no apparent change concerning the size of motor neuron columns after 48 h of forced CyclinD1 or D2 expression (Figure 4b–l), the spinal ventral root was larger on the transgenic side compared to the contralateral control side after 72 h of forced CyclinD1 expression (Figure 6b,d). We quantified the spinal ventral root by measuring the span of the ventral root at the emerging point on each side and determining the ratio of the transgenic side/control side (Figure 6d, dashed lines). We measured an increase in this ratio following forced expression of CyclinD1 compared to that found in embryos electroporated with a control vector; 1.6 ± 0.31 (n = 7 sections from 3 embryos) compared to 0.99 ± 0.17 (n = 4 sections), respectively. This observation suggests an increase in the number of motor neurons extending axons in the ventral horn following 72 h of CyclinD forced expression. To determine any augmentation in the motor neuron population, we quantified and compared the number of Islet1/2-positive cells on the control side versus the CyclinD1 electroporated side (Figure 6g,h). The ratio of Islet1/2+ cells on the transgenic side versus the control side was 1.59 ± 0.03 (SEM, n = 42 optical sections from 3 embryos). These data confirm an increase in the number of differentiating motor neurons following 72 h of CyclinD1 forced expression and suggest that cycling and differentiating motor neurons augment the neuronal population.

Further analysis of the GFP+ cells located in the Islet1/2+ domain revealed that all the transgenic cells co-expressed the pan-motor neuron marker Islet1/2. Moreover, Lim1/2, a widely expressed marker of interneurons, remained excluded from the transgenic population located in the motor neuron columns (data not shown). These observations suggest that the dividing cells located in the ventral horn gave rise to motor neurons.

### Sustained expression of CyclinD allows only a limited number of divisions in the motor neuron population

We next wondered whether forced expression of CyclinD1 would cause differentiating motor neurons to keep cycling for a number of divisions or if the effect was transient. To gain insight into this we determined the proliferation rate in the GFP+/Islet1/2+ cells after 72 h of forced CyclinD1 expression (Figure 6i,j) and compared it to that observed at 48 h (Figure 4l,m). After a 30 minute pulse of BrdU, only 11% ± 1.21 (SEM, n = 19 optical sections from 3 embryos) of transgenic cells incorporated BrdU (versus 32% ± 3.5 at 48 h), indicating a rapid decrease in the proliferation rate in the differentiating motor neurons. One feasible explanation for this could be the loss of transgenic CyclinD from these differentiating motor neurons; however, the use of the tagged version of CyclinD2 clearly demonstrated a remaining considerable co-expression of the transgene with the GFP, thereby arguing against such a possibility (Figure 6k,l).

Another explanation for the decrease may be that the proliferating cells undergo apoptosis, thus explaining the rapid loss of the proliferating population. To investigate this possibility, we determined the level of an active form of caspase 3 in the spinal cord at 72 h or 96 h post electroporation of CyclinD1. We detected no significant difference in caspase 3 levels (data not shown), suggesting that differentiating motor neurons perform only a limited number of divisions despite the maintenance of CyclinD.

### Influence of neuroepithelium age on the behavior of neural progenitor cells

**Figure 7:**
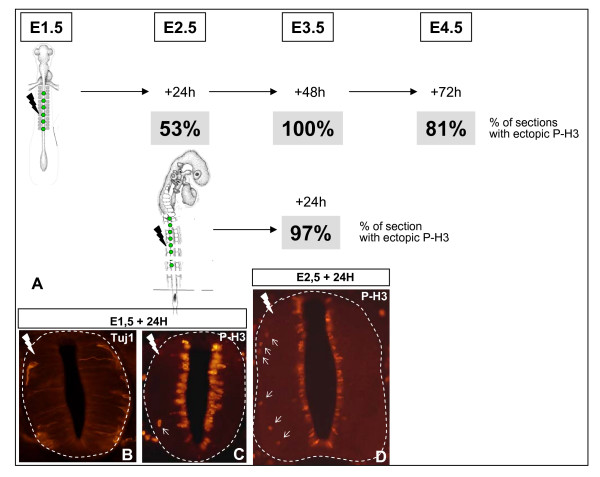
**Influence of the maturity of the neural tube on phenotype occurrence.(a) **Schematic representation showing the age of the embryo at the time of electroporation and the different times after electroporation at which the embryos were fixed to evaluate the phenotype. For each experimental condition, 23 to 48 sections from 2 to 3 electroporated embryos were analyzed and the percentage of sections displaying ectopic P-H3 cells on the transgenic side determined. The corresponding values are reported for each experimental condition. **(b-d) **Cross-sections of the spinal cord showing the phenotype 24 h after electroporation at E1.5 (b,c) and E2.5 (d), respectively. Tuj1 (b) marks the differentiating neurons. The arrows in (c,d) mark mitotic cells in ectopic positions. (b-d) Images obtained with an epifluorescent microscope.

We previously observed that overexpressing CyclinD for 24 h in the neural tube of 1.5-day-old embryos impedes neuronal differentiation while a further 24 h (total 48 h) leads to differentiating and cycling cells. Two hypotheses can be proposed: the critical factor is either the length of CyclinD overexpression (24 h versus 48 h) or the age of the neuroepithelium (E2.5 and E3.5, respectively, at the time of analyses). To discriminate between these hypotheses, we electroporated the neural tube of 1.5- and 2.5-day-old embryos and evaluated the phenotype by quantifying the number of transgenic sections displaying ectopic P-H3 staining in the mantle layer 24 h and 48 h following overexpression at E1.5 and 24 h after overexpression at E2.5; the data are illustrated in Figure 7. At 48 h after electroporation at E1.5, 100% of the sections displayed an identical phenotype to that obtained at 24 h following electroporation at E2.5 (97%). Conversely, only half of the sections displayed this phenotype 24 h after electroporation at E1.5 (53%). The same evaluation of the phenotype performed 72 h after electroporation at E1.5 revealed 81% of the sections still displaying P-H3 positive cells. These data point more to an influence of age of the neural tube than to the length of CyclinD forced expression on the phenotype, and that the most penetrant phenotype is indeed observed at the peak of motor neuron production (E3.5).

## Discussion

### Cell cycle and neurogenesis

One intriguing observation we made is the domain-specific expression of CyclinD1 and D2 in the chicken neural tube. The pMNs express mostly CyclinD1, suggesting that this cell cycle regulator might play a critical role in the maintenance of this particular progenitor domain. Concordantly, a recent study reported that the pMNs of *Xenopus *embryos also specifically express one CyclinD, related to CyclinD1, and that inhibition of this CyclinD results in the specific loss of the pMNs [20]. In our gain-of-function experiments, CyclinD1 and CyclinD2 behaved identically in their ability to maintain transgenic cells in the cell cycle even during differentiation. This suggests some redundancy in their function; however, our strategy did not allow us to ascertain their possible subtle functional differences. Such differences have recently been reported in the embryonic brain with CyclinD1 and CyclinD2 defining separate progenitor pools [21,22]. Comparative analysis of cell cycle regulation in D1- and D2-null mice suggests that compared to CyclinD1, CyclinD2 exerts a stronger inhibitory influence on p27 to delay the cell cycle exit of progenitors [22].

One important characteristic that applies to all mature neurons is that they never divide. Nevertheless, data have accumulated showing that young neurons undergo extra divisions when CKIs are removed [10,17]. These molecules are known to enforce permanent cell cycle exit upon terminal differentiation. In mice lacking the CKIs p19/Ink4d and p27/kip1, neurons divide after migration to their final position in the brain [17]. In the absence of p57Kip2, many spinal interneurons re-enter the cell cycle for at least one additional round of cell division before ultimately differentiating [10]. Indeed, p57Kip2 has been shown to arrest cell cycle progression at G1 by antagonizing CyclinD1, probably among other activities. As yet, p27 is the only CKI found to be expressed in young post-mitotic motor neurons, although p27-/- embryos have no detectable neurogenic defect [10]. One remaining open question is whether cell cycle exit is a prerequisite for motor neuron differentiation. Here we have shown that, as with interneurons, motor neurons can migrate into the differentiating field and differentiate without exiting the cell cycle. This phenotype culminates at the peak of neuronal production and results in an increase in the number of motor neurons, as is observed for interneurons. Moreover, our results demonstrate the ability of neurons to upregulate a program of transcription factors associated with early differentiation and extend axons while cycling.

### Controlling cell cycle exit in pMNs

This now leads on to questions surrounding the possible mechanism controlling the cell cycle exit for motor neurons. Although p27 seems dispensable for cell cycle exit in motor neurons, other molecules besides those of the CKI family could be involved in controlling cell cycle exit for this particular neuronal population. A recent study performed in *Drosophila *proposed that both the retinoblastoma and p27 homologs contribute to parallel repression of E2F and Cyclin/Cdk activity, respectively, in differentiating neurons [23]. Interestingly, in mouse embryos in which the retinoblastoma protein is inactivated, many ectopically dividing cells are found in the central and peripheral nervous system, including the ventral horns of the spinal cord where motor neurons differentiate [15,16]. This suggests that the retinoblastoma protein (pRB) may also play an important repressive role in motor neuron differentiation-associated cell cycle exit. One reasonable hypothesis is that overexpression of CyclinD in the neural tube gives rise to ectopic mitosis, in part by maintaining pRB in an inactive hyperphosphorylated state, thus phenocopying the phenotype of pRB loss-of-function.

### Forcing neural progenitor cells to cycle is not sufficient to alter cell-fate choice

We have shown that keeping a neural progenitor cycling is not sufficient to maintain them in an undifferentiated state in reserve for later born cell types. Indeed, forced proliferation of the progenitors of the pMN domain, which first produce motor neurons and, later on, oligodendrocytes, did not abolish motor neuron production but instead gave rise to cycling differentiating motor neurons. Furthermore, we detected no change at the onset of oligodendrocyte production (data not shown). One unanswered question concerns the fate of the daughter cells of the dividing motor neurons. While only a clonal analysis experiment may answer this question accurately, certain observations suggest that they give rise to two motor neurons: firstly, they are unlikely to die rapidly after division since we detected no increase in cell death in the transgenic population; secondly, 72 h following electroporation, the number of differentiating motor neurons was found to be higher in the population containing dividing transgenic cells when compared to the contralateral control side, with all the transgenic cells expressing the pan-motor neuron marker Islet1/2. It could be argued that the increase in the number of differentiating motor neurons is the consequence of an increase in the population of pMNs in transgenic embryos. However, the size of the Olig2+ progenitor domain showed no significant increase following forced expression of CyclinD1, suggesting that the rise in motor neuron population is rather the consequence of dividing differentiating motor neurons than an increase in the progenitor population. Nevertheless, we cannot exclude that the increase in the differentiating motor neuron population occurs, at least in part, as a consequence of an increased production of neural progenitor cells that rapidly migrate into the differentiating field and initiate their differentiation without exiting the cell cycle.

### Controlling the timing of neuronal differentiation

Our data argue against cell cycle exit being the gateway in the timing of motor neuron differentiation; however; it is still possible that other cell cycle parameters influence this timing event. Contrary to what has been reported for cortical development [24], the orientation of cell division does not seem to be a critical factor in timing neurogenesis in the spinal cord [25-28]. One promising possibility is the effect of varying cell cycle kinetics, including the duration and length of the gap phases, which have been shown to influence the switching of neural progenitors from proliferative to neuron-generating divisions in the retina and brain [29-32]. Concordantly, in the chicken neural tube, fibroblast growth factor-mediated inhibition of neuronal differentiation is associated with cell cycle acceleration [28].

In our experimental context, we observed cycling differentiated neurons in the mantle layer. Examples of cells retaining neural progenitor characteristics found in the mantle layer have also been described previously. Jmj is a direct transcriptional repressor of CyclinD1 and ectopic proliferating cells are present in the mantle layer in the hindbrain of *jmj *mutant mice [14]. Moreover, crossing *jmj *mutants with mice in which CyclinD1 function has been removed is sufficient to rescue the phenotype. According to our observations, we would expect that the cycling cells in the mantle layer of *jmj *mutants are differentiating neurons. On the contrary, the ectopic cycling cells in *jmj *mutants were shown to retain markers of the ventricular zone without expressing markers of differentiation [14]. The easiest interpretation of this apparent paradox is a difference in the behavior of neural progenitor cells in the hindbrain compared to the spinal cord, in agreement with the absence of ectopic proliferating cells in the spinal cord of the *jmj *mutant. The presence of proliferating cells retaining traits of neural progenitors, including Sox2 expression, in the spinal mantle layer has been reported following inhibition of the G-protein regulator LNG [27]. LNG regulates mitotic spindle movements to favor planar division, giving rise to two progenitor cells. Inhibition of LNG activity leads to random spindle orientation, resulting in the neural progenitor cells leaving the ventricular zone prematurely though retaining neural progenitor characteristics whilst in the differentiating field. This suggests that these cells do not actually reach the maturation state enabling them to differentiate further in the mantle layer. Hence, in addition to cell cycle exit, the maturation state of the neural progenitor cells would influence the timing of neuronal differentiation. In accordance with this, we observed that while forced expression of CyclinD in the young neural tube promotes progenitor cell proliferation at the expense of neuronal differentiation [13], this phenotype is transient and neural progenitor cells soon afterwards migrate into the mantle layer and differentiate, regardless of their proliferation status. What changes occur between a young and more mature neural tube to explain such a difference? One of the major differences occurring during the maturation of the neuroepithelium is the setting-up of combinations of homeodomain proteins into discrete progenitor cell domains [1]. Neural progenitor cells of a particular domain are then destined to give rise to a particular subtype of neurons. Furthermore, these patterning genes not only direct cell identity but also play a key role in controlling the timing of neuronal differentiation. Thus, in the pMN, Olig2 upregulates the bHLH factor Ngn2, and the ratio Olig2/Ngn2 controls motor neuron differentiation [3,4,6]. Pax6 also pushes neural progenitor cells towards neuronal commitment via Ngn2 upregulation [33-36], with the switching off of Pax6 being required for the initiation of neuronal differentiation [33]. Our data show that in cells overexpressing CyclinD, upregulation of Ngn2 takes place and the temporal switching off of Olig2 and Pax6 occurs as committed neurons leave the ventricular zone, leading to the differentiation of the neurons regardless of cell cycle exit. Together, these data strongly suggest that while cell cycle exit is not necessary for differentiation, the switching off of patterning genes is a determining event in the control of the timing of neuronal differentiation, at least in the spinal cord.

## Conclusion

The present study, using the pMN as a model, demonstrates that forcing a neural progenitor cell to cycle is not sufficient to keep it undifferentiated in the spinal cord. Instead, the neural progenitor cells were shown to differentiate into neurons and project axons into the right pathways, regardless of cell cycle exit. We propose that once the combinatorial expression of homeodomain proteins is set-up in a spinal progenitor domain, this program is robust enough to promote the activation of the transcriptional sequence involved in the specification and differentiation of neurons independently of cell cycle exit. Therefore, cell cycle exit seems not to be a prerequisite to neuronal differentiation in the formation of post-mitotic neurons; rather, both events are coordinated during normal neurogenesis.

## Materials and methods

### Embryos

Fertile hens' eggs, obtained from a local supplier, were incubated at 38°C in a humidified incubator to yield embryos of appropriate stages [37].

### DNA constructs and *in ovo *electroporation

*In ovo *electroporation experiments were performed using pCIG-CyclinD1-IRES-GFP or pCIG-CyclinD2-IRES-GFP constructs as already described [13]. To visualize the axons of transgenic cells, the nuclear GFP from the pCIG-CyclinD1-IRES-GFP was replaced by eGFP. Chicken CyclinD2 was cloned into the pTracer-EF (Invitrogen, Carlsbad, CA, USA) containing a V5 tag and the tagged cDNA subcloned into pCIG [38]. Vectors were electroporated as previously described [13].

### *In situ *hybridization and immunohistochemistry

Detection of transcripts and proteins were performed on 40 μm vibratome sections as previously described [13]. The antibodies used in the present studies were: anti-Nkx2.2, anti-Pax7, anti-BrdU, anti-Islet1/2, anti-MNR2, anti-Lim1/2 and anti-BEN/SC1/DM-GRASP (Hybridoma Bank), anti-Sox2 (a gift from Dr T Edlund), anti-P-H3 (Upstate Biotechnology, Lake Placid, NY, USA), O4 antibody (a gift from Dr R Bansal), anti-HuC/D (Molecular Probes, Eugene, OR, USA), anti-GFP (Torrey Pines Biolabs, Houston, TX, USA), anti-Olig2 (Chemicon, Billerica, MA, USA), anti-active caspase-3 (BD PharMingen, Franklin Lakes, NJ, USA), anti-Pax6 (BAbCO, Richmond, CA, USA), anti-Ngn2 (a gift from D Anderson [5]), anti-neuronal class III β-tubulin (Tuj1, BAbCO), and anti-V5 (Invitrogen).

### Cell proliferation analysis

Cell proliferation was evaluated by BrdU incorporation (BrdU labeling and detection kit I, Roche, Basel, Switzerland). BrdU (10 mM) was injected into the lumen of the neural tube and the embryos harvested 30 minutes later. BrdU immunodetection was performed on vibratome sections as previously described [13].

### Analysis and imaging of the data

Sections of 40 μm were analyzed using an epifluorescent Nikon microscope or a SP2 Leica confocal microscope. Confocal analyses of the 40 μm vibratome sections were performed on sections acquired at 5 μm Z steps. The data are presented as the maximum projection of optical sections or as single optical sections, as mentioned in the figure legends.

## Competing interests

The author(s) declare that they have no competing interests.

## Authors' contributions

VL participated in the design of the study, performed overexpression experiments and immunohistochemistry, analyzed and quantified the data, and helped to draft the manuscript. SBV participated in the design of the study, performed overexpression experiments and immunohistochemistry, obtaining all the data at 72 h, and helped to draft the manuscript. FT performed part of the overexpression experiments and immunohistochemistry performed at 48 h and helped in critical reading of the manuscript. FP conceived the study and participated in its design and coordination as well as drafting the manuscript. All authors read and approved the final manuscript.
